# Comparative analysis of microwave radiation generated by the interaction between electron beam and plasma under different methods

**DOI:** 10.1038/s41598-024-74973-6

**Published:** 2024-10-07

**Authors:** Shen Gao, Jing-Xin Liu, Jin-Ke Zhang

**Affiliations:** School of Electrical and Information, ChangZhou Institute of Technology, ChangZhou, 213032 China

**Keywords:** Electron beam, Plasma, Microwave radiation, Dispersion relation, Plasma physics, Computational science

## Abstract

This article presents a physical model that describes the interaction between surface electron beams and plasma. The dispersion relations for beam plasma interactions were derived using perturbation method and field matching methods. The study investigates how different parameters affect radiation frequency and bandwidth. The results indicate that as electron beam velocity increases, the associated kinetic energy also rises, leading to an increase in both the maximum radiation frequency and bandwidth at high frequencies. Conversely, the radiation bandwidth at low frequencies decreases. Similarly, a higher plasma density results in a greater maximum radiation frequency, but the high-frequency bandwidth decreases, while the low-frequency bandwidth increases. Additionally, when the electron density and electron velocity of the electron beam remain constant, increasing the plasma density can increase the microwave radiation frequency However, there exists a plasma density threshold, beyond which high-frequency electromagnetic waves are no longer radiated.

## Introduction

The interaction between electron beam and plasma can generate wide-band microwave radiation^1–4^. Plasma radiation sources have the following characteristics^5–7^: (1) The presence of background plasma neutralizes the beam current, allowing the system to overcome the extreme current limitations found in vacuum devices and enhancing the interaction between the beam and electromagnetic waves. (2) Broadband radiation can be achieved by adjusting the ratio between beam and plasma densities. (3) The radiation frequency can be tuned by altering the plasma density.

Normally, in experiments involving the interaction between electron beams and plasma, the electron beam is a cylindrical structure. This article uses a surface electron beam. Considering the increase in the contact surface between the electron beam and the plasma, under the same current density, does it increase the power or frequency of microwave radiation. Therefore, before conducting relevant experiments, this article first derived and analyzed its dispersion relationship.

The mechanism behind beam-plasma interactions that generate broadband microwave radiation is complex. Typically, either the perturbation method or the field matching method is used to solve the dispersion relation. In the early research, the dielectric tensor of plasma was directly introduced into Maxwell’s equations, and the dispersion relation of the plasma was solved using the field matching method. However, this approach only considers the one-dimensional longitudinal effects and neglects the influence of electromagnetic fields on the plasma itself^8^. The second approach combines the equation of motion for electron beam with Maxwell’s equation, using the perturbation method to solve the dispersion relation, which is influenced by the factors such as electron beam density, electron velocity, and the applied magnetic field. However, this method does not account for the mutual interaction between the electron beam and plasma, nor does it consider the interaction between microwaves and plasma^9^. On one hand, the electron beam and space electromagnetic fields influence the plasma density distribution, while on the other hand, the plasma density in turn affects the microwave radiation frequency.

Bothe the perturbation method and the field matching method are commonly used to solve the dispersion relationship of plasma. The perturbation method is relatively simple to solve and usually considers the plasma in an infinite range. On the other hand, the field matching method takes into account the boundary conditions between the electron beam and the plasma. Compared with perturbation method, the field matching method provides higher accuracy. However, due to its inherent characteristics, the field matching method can only solve the relationship between the wave vector *k*_*z*_ and the frequency *w*, without analyzing the radiation direction of the electromagnetic field. Taking these factors into consideration this paper establishes a more accurate mathematical model for beam-plasma microwave radiation. In the second part of this article, the perturbation method is used to solve the dispersion relationship for the interaction between electron beams and plasma. The relationship between *k*_z_ and *w* is analyzed based on the above results. The third part of this article employs the field matching method to solve the dispersion relationship for the electron beam-plasma interaction. This not only verifies the correctness of the results obtained via the perturbation method but also analyzes the effects of radiation bandwidth and various parameters.

## Solution of dispersion relation - perturbation method

**Fig. 1:**
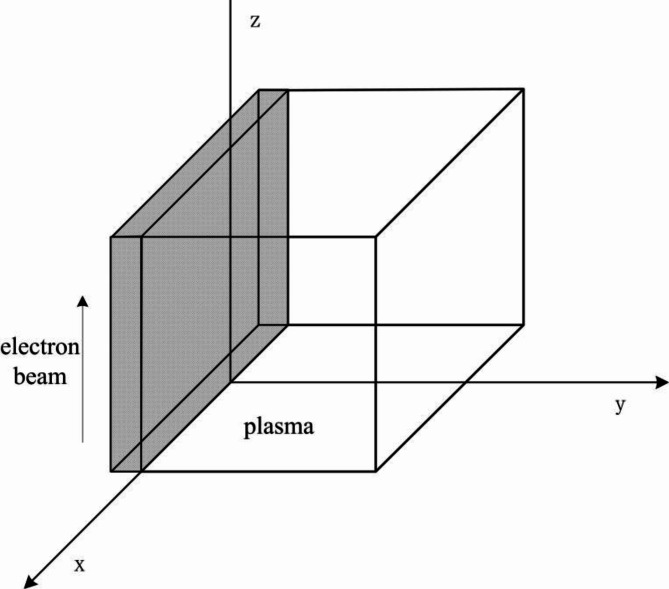
Physical model of plasma microwave radiation.

The physical model of plasma microwave radiation is illustrated in Fig. 1, where a surface electron beam interacts with plasma. The electron beam moves in the z-direction. The physical model shown in Fig. 1 only represents the model of the electron beam and plasma at the initial moment, without depicting the disturbance process during the interaction between the electron beam and plasma. In fact, their boundaries resemble the oscillation form of waves, which are represented by straight lines for simplicity. The physical model in the figure mainly indicates the relative position in the coordinate system.

We focus on the transverse magnetic wave propagating perpendicular to the direction of the induced magnetic field, $\vec {k} \cdot {B_x}=0$, ${B_x}$ is the induced magnetic field generated by beam plasma interaction. Assume that the direction of ${B_x}$ is the x direction, so the electromagnetic wave has two components: ${E_y}$ and ${E_z}$, so it can be set:

1 $${E_{1y}}{\text{=}}{E_y}{e^{i\left( {{{\overset{\lower0.5em\hbox{$\smash{\scriptscriptstyle\rightharpoonup}$}} {k} }_y} \cdot \overset{\lower0.5em\hbox{$\smash{\scriptscriptstyle\rightharpoonup}$}} {y} +{{\overset{\lower0.5em\hbox{$\smash{\scriptscriptstyle\rightharpoonup}$}} {k} }_z} \cdot \overset{\lower0.5em\hbox{$\smash{\scriptscriptstyle\rightharpoonup}$}} {z} - \omega t} \right)}}$$

2 $${E_{1z}}{\text{=}}{E_z}{e^{i\left( {{{\overset{\lower0.5em\hbox{$\smash{\scriptscriptstyle\rightharpoonup}$}} {k} }_y} \cdot \overset{\lower0.5em\hbox{$\smash{\scriptscriptstyle\rightharpoonup}$}} {y} +{{\overset{\lower0.5em\hbox{$\smash{\scriptscriptstyle\rightharpoonup}$}} {k} }_z} \cdot \overset{\lower0.5em\hbox{$\smash{\scriptscriptstyle\rightharpoonup}$}} {z} - \omega t} \right)}}$$

3 $${B_1}{\text{=}}{B_{1x}}\overset{\lower0.5em\hbox{$\smash{\scriptscriptstyle\rightharpoonup}$}} {x} {e^{i\left( {{{\overset{\lower0.5em\hbox{$\smash{\scriptscriptstyle\rightharpoonup}$}} {k} }_y} \cdot \overset{\lower0.5em\hbox{$\smash{\scriptscriptstyle\rightharpoonup}$}} {y} +{{\overset{\lower0.5em\hbox{$\smash{\scriptscriptstyle\rightharpoonup}$}} {k} }_z} \cdot \overset{\lower0.5em\hbox{$\smash{\scriptscriptstyle\rightharpoonup}$}} {z} - \omega t} \right)}}$$

When discussing the weak interaction of the electron beam and the plasma, we assume that the ions remain stationary. The relevant physical quantities can be expressed as constants in the equilibrium state plus varying perturbations, that is$f={f_0}+{f_1}$. Subscript 0 represents the quantity in the equilibrium state, subscript 1 represents the disturbance quantity, disturbance quantity is ${f_1} \propto \exp \left( {i{k_z}z+i{k_y}y - i\omega t} \right)$, and ${f_0} \gg {f_1}$, ${k_z},{k_y}$ and $\omega$ are wave number and angular frequency, respectively.

The model above can be viewed as the interaction between two types of plasma. The electron beam can be regarded as a non-neutral plasma in high-speed motion, and therefore it adheres to the laws of particle number conservation and momentum conservation^10–12^. Due to the relativistic velocity of the electron beam, a relativistic factor is introduced in Eq. 7. Similarly, the plasma also follows the equations of particle number conservation and momentum conservation. Compared with high-energy electron beams, the initial velocity of the plasma is assumed to be 0. The ion term equations (including ion number momentum conservation and particle number conservation) in the plasma are neglected, as ions mainly generate ion acoustic waves. Equations 8 and 9 represent Maxwell’s equations, while Eq. 10 describes the current density generated by electrons.

4 $$\frac{{\partial {n_e}}}{{\partial t}}+\nabla \cdot \left( {{n_e}{u_e}} \right)=0$$

5 $${m_e}{n_e}\left\{ {\frac{\partial }{{\partial t}}+{u_e} \cdot \nabla } \right\}{u_e}= - e{n_e}\left( {E+{u_e} \times B} \right)$$

6 $$\frac{{\partial {n_b}}}{{\partial t}}+\nabla \cdot \left( {{n_b}{u_b}} \right)=0$$

7 $${\gamma _0}{m_e}\left\{ {\frac{\partial }{{\partial t}}+{u_b} \cdot \nabla } \right\}{u_b}+{m_e}{u_b}\frac{{\partial {\gamma _1}}}{{\partial t}}= - e\left( {E+{u_b} \times B} \right)$$

8 $$\nabla \times {\overset{\lower0.5em\hbox{$\smash{\scriptscriptstyle\rightharpoonup}$}} {E} _i}= - \frac{{\partial {{\overset{\lower0.5em\hbox{$\smash{\scriptscriptstyle\rightharpoonup}$}} {B} }_i}}}{{\partial t}}$$

9 $$\nabla \times {\overset{\lower0.5em\hbox{$\smash{\scriptscriptstyle\rightharpoonup}$}} {B} _i}={\mu _0}J+\frac{1}{{{C^2}}}\frac{{\partial {{\overset{\lower0.5em\hbox{$\smash{\scriptscriptstyle\rightharpoonup}$}} {E} }_i}}}{{\partial t}}$$

10 $${\mathbf{J}}{\text{=}}\left( { - e{n_{b0}}{u_{bz}} - e{n_{b1}}{u_{b0z}} - e{n_{p0}}{u_{ez}}} \right)\overset{\lower0.5em\hbox{$\smash{\scriptscriptstyle\rightharpoonup}$}} {z} +\left( { - e{n_{p0}}{u_{ey}} - e{n_{b0}}{u_{by}}} \right)\overset{\lower0.5em\hbox{$\smash{\scriptscriptstyle\rightharpoonup}$}} {y}$$

Where ${n_e}$ is the plasma electron density; ${n_b}$ is the electron beam density; ${u_e}$ is the plasma electron velocity; ${u_b}$is the electron beam velocity; ${m_e}$is the electronic quality; $\gamma$ is the relativistic factor, ${{\gamma =1} \mathord{\left/ {\vphantom {{\gamma =1} {\sqrt {1 - {{u_{b}^{2}} \mathord{\left/ {\vphantom {{u_{b}^{2}} {{C^2}}}} \right. \kern-0pt} {{C^2}}}} }}} \right. \kern-0pt} {\sqrt {1 - {{u_{b}^{2}} \mathord{\left/ {\vphantom {{u_{b}^{2}} {{C^2}}}} \right. \kern-0pt} {{C^2}}}} }}$and *C* is speed of light, because the speed of the electron beam is in the following form: ${u_b}={u_{b0z}}+{u_{b1}}$, the relativistic factor can therefore also be expanded as follows:$\gamma ={\gamma _0}+{\gamma _1}+{R_n}\left( {{u_{b1}}} \right)$,${\gamma _1}$ is a first-order Taylor expansion at position ${u_{b1}}$; ${R_n}\left( {{u_{b1}}} \right)$ is a high-order infinitesimal; ***E*** is the induced electric field; ***B*** is is the total magnetic induction, including the magnetic field generated by the electron beam ${B_b}$ and the induced magnetic field ${B_1}$.

According to Ampere’s loop law, the magnetic field in the electron beam is

11 $${B_b}= - {{{\mu _0}e{n_{b0}}{u_{b0}}y} \mathord{\left/ {\vphantom {{{\mu _0}e{n_{b0}}{u_{b0}}y} 2}} \right. \kern-0pt} 2}\left( {y \leqslant {y_0}} \right)$$

Where *y*_0_ represents the boundary of the electron beam or the thickness of the electron beam. The derivative of *y* with respect to time can be expressed as

12 $$\frac{{dy}}{{dt}}=\left( {\frac{\partial }{{\partial t}}+{u_{b0}}\frac{\partial }{{\partial z}}} \right)y={u_{by}}$$

Inside the electron beam, the magnetic field generated by the electron beam is

13 $${B_b}=\frac{{{\mu _0}e{n_{b0}}{u_{b0}}}}{{2i\left( {\omega - {k_z}{u_{b0}}} \right)}}{u_{by}}$$

Inside the electron beam,${u_{b1}} \times {B_{b0}}$is a higher order small quantity, which can be ignored. Therefore, within the electron beam, the influence of the x-direction magnetic field generated by the electron beam itself is neglected; while outside the electron beam (at the plasma), the magnetic field generated by the electron beam is not neglected.

For the middle term of Eq. (7)

14 $${m_e}{u_b}\frac{{\partial {\gamma _1}}}{{\partial t}}=\frac{{{u_b}}}{{{C^2}}}\frac{{d\left( {\gamma {m_e}{C^2}} \right)}}{{dt}}=\frac{{{u_b}}}{{{C^2}}}\frac{{dW}}{{dt}}$$

*W* is the energy of an electron. Because

15 $$\frac{{dW}}{{dt}}=F \cdot {u_b}=e\left( {E+{u_b} \times B} \right) \cdot {u_b}$$

Substituting Eqs. (15) and (14) into Eq. (7) yields

16 $${\gamma _0}{m_e}\left\{ {\frac{\partial }{{\partial t}}+{u_b} \cdot \nabla } \right\}{u_b}+\frac{{{u_b}}}{{{C^2}}}eE \cdot {u_b}= - e\left( {E+{u_b} \times B} \right)$$

After perturbing Eqs. 4–10, omitting high-order small quantities of order 2 or higher, the following equation system is obtained

17 $$\left\{ \begin{gathered} - \omega {n_{p1}}+{k_y}{n_{p0}}{u_{ey}}+{k_z}{n_{p0}}{u_{ez}}=0 \hfill \\ i\omega {m_e}{n_{p0}}{u_{ey}}=e{n_{p0}}{E_{1y}}+e{n_{p0}}{u_{ez}}{B_b} \hfill \\ i\omega {m_e}{n_{p0}}{u_{ez}}=e{n_{p0}}{E_{1z}} - e{n_{p0}}{u_{ey}}{B_b} \hfill \\ \left( {{k_z}{u_{b0z}} - \omega } \right){n_{b1}}+{k_y}{n_{b0}}{u_{by}}+{k_z}{n_{b0}}{u_{bz}}=0 \hfill \\ {\gamma _0}{m_e}\left( { - i\omega +i{k_z}{u_{b0z}}} \right){u_{bz}}= - \left( {{{u_{{b0z}}^{2}} \mathord{\left/ {\vphantom {{u_{{b0z}}^{2}} {{C^2}}}} \right. \kern-0pt} {{C^2}}}+1} \right)e{E_{1z}} \hfill \\ {\gamma _0}{m_e}\left( { - i\omega +i{k_z}{u_{b0z}}} \right){u_{by}}= - e{E_{1y}} - e{u_{b0z}}{B_{1x}} \hfill \\ {k_y}{E_{1z}} - {k_z}{E_{1y}} - \omega {B_{1x}}{\text{=0}} \hfill \\ {n_{b0}}{u_{by}}+{n_{p0}}{u_{ey}}+{{i\omega {E_{1y}}} \mathord{\left/ {\vphantom {{i\omega {E_{1y}}} {{\mu _0}e{C^2}}}} \right. \kern-0pt} {{\mu _0}e{C^2}}}+{{i{k_z}{B_{1x}}} \mathord{\left/ {\vphantom {{i{k_z}{B_{1x}}} {{\mu _0}e}}} \right. \kern-0pt} {{\mu _0}e}}=0 \hfill \\ {n_{b1}}{u_{b0z}}+{n_{b0}}{u_{bz}}+{n_{p0}}{u_{ez}}+{{i\omega {E_{1z}}} \mathord{\left/ {\vphantom {{i\omega {E_{1z}}} {{\mu _0}e{C^2}}}} \right. \kern-0pt} {{\mu _0}e{C^2}}} - {{i{k_y}{B_{1x}}} \mathord{\left/ {\vphantom {{i{k_y}{B_{1x}}} {{\mu _0}e}}} \right. \kern-0pt} {{\mu _0}e}}=0 \hfill \\ \end{gathered} \right.$$

Using Eqs. 2 and 3 in Eq. 17, the plasma electron velocity can be solved

18 $$\left\{ \begin{gathered} {u_{ey}}=\frac{1}{{\left[ {1+{{\left( {\frac{{e{B_b}}}{{i\omega {m_e}}}} \right)}^2}} \right]}}\frac{e}{{i\omega {m_e}}}{E_{1y}}+\frac{1}{{\left[ {1+{{\left( {\frac{{e{B_b}}}{{i\omega {m_e}}}} \right)}^2}} \right]}}{\left( {\frac{e}{{i\omega {m_e}}}} \right)^2}{B_b}{E_{1z}} \hfill \\ {u_{ez}}=\frac{1}{{\left[ {1+{{\left( {\frac{{e{B_b}}}{{i\omega {m_e}}}} \right)}^2}} \right]}}\frac{e}{{i\omega {m_e}}}{E_{1z}} - \frac{1}{{\left[ {1+{{\left( {\frac{{e{B_b}}}{{i\omega {m_e}}}} \right)}^2}} \right]}}{\left( {\frac{e}{{i\omega {m_e}}}} \right)^2}{B_b}{E_{1y}} \hfill \\ \end{gathered} \right.$$

Using Eq. 7 in Eq. 17 to solve linear relationship between induced magnetic field and electric field components

19 $${B_{1x}}=\frac{{{k_y}}}{\omega }{E_{1z}} - \frac{{{k_z}}}{\omega }{E_{1y}}$$

Using Eqs. 5and 6 in Eq. 17, and Eq. 19 to solve the electron beam electron velocity

20 $$\left\{ \begin{gathered} {u_{bz}}=\frac{{e\left( {{{u_{{b0z}}^{2}} \mathord{\left/ {\vphantom {{u_{{b0z}}^{2}} {{C^2}}}} \right. \kern-0pt} {{C^2}}}+1} \right)}}{{{\gamma _0}{m_e}\left( {i\omega - i{k_z}{u_{b0z}}} \right)}}{E_{1z}} \hfill \\ {u_{by}}=\frac{e}{{{\gamma _0}{m_e}\left( {i\omega - i{k_z}{u_{b0z}}} \right)}}{E_{1y}} - \frac{{e{u_{b0z}}}}{{{\gamma _0}{m_e}\left( {i\omega - i{k_z}{u_{b0z}}} \right)}}\frac{{{k_z}}}{\omega }{E_{1y}}+\frac{{e{u_{b0z}}}}{{{\gamma _0}{m_e}\left( {i\omega - i{k_z}{u_{b0z}}} \right)}}\frac{{{k_y}}}{\omega }{E_{1z}} \hfill \\ \end{gathered} \right.$$

Substituting Eq. 20 into Eq. 17 with Eq. 4

21 $$\begin{gathered} {n_{b1}}=\frac{1}{{{k_z}{u_{b0z}} - \omega }}\left[ {\frac{{e{k_y}{n_{b0}}{u_{b0z}}}}{{{\gamma _0}{m_e}\left( {i\omega - i{k_z}{u_{b0z}}} \right)}}\frac{{{k_z}}}{\omega } - \frac{{e{k_y}{n_{b0}}}}{{{\gamma _0}{m_e}\left( {i\omega - i{k_z}{u_{b0z}}} \right)}}} \right]{E_{1y}} \\ - \frac{1}{{{k_z}{u_{b0z}} - \omega }}\left[ {\frac{{e{k_y}{n_{b0}}{u_{b0z}}}}{{{\gamma _0}{m_e}\left( {i\omega - i{k_z}{u_{b0z}}} \right)}}\frac{{{k_y}}}{\omega }+\frac{{e{k_z}{n_{b0}}\left( {{{u_{{b0z}}^{2}} \mathord{\left/ {\vphantom {{u_{{b0z}}^{2}} {{C^2}}}} \right. \kern-0pt} {{C^2}}}+1} \right)}}{{{\gamma _0}{m_e}\left( {i\omega - i{k_z}{u_{b0z}}} \right)}}} \right]{E_{1z}} \\ \end{gathered}$$

Substituting Eqs. 18, 19, 20, 21 into Eq. 17 with Eqs. 8 and 9, we obtained

22 $${A_{11}}{E_{1y}}+{A_{12}}{E_{1z}}=0$$

23 $${A_{21}}{E_{1y}}+{A_{22}}{E_{1z}}=0$$

Where

24 $$\left\{ \begin{gathered} {A_{11}}=\frac{{e{n_{b0}}}}{{{\gamma _0}{m_e}\left( {i\omega - i{k_z}{u_{b0z}}} \right)}} - \frac{{e{n_{b0}}{u_{b0z}}}}{{{\gamma _0}{m_e}\left( {i\omega - i{k_z}{u_{b0z}}} \right)}}\frac{{{k_z}}}{\omega }+\frac{{{n_{p0}}}}{{\left[ {1+{{\left( {\frac{{e{B_b}}}{{i\omega {m_e}}}} \right)}^2}} \right]}}\frac{e}{{i\omega {m_e}}}+\frac{{i\omega }}{{{\mu _0}e{C^2}}} - \frac{{i{k_z}}}{{{\mu _0}e}}\frac{{{k_z}}}{\omega } \hfill \\ {A_{12}}=\frac{{e{n_{b0}}{u_{b0z}}}}{{{\gamma _0}{m_e}\left( {i\omega - i{k_z}{u_{b0z}}} \right)}}\frac{{{k_y}}}{\omega }+\frac{{{n_{p0}}}}{{\left[ {1+{{\left( {\frac{{e{B_b}}}{{i\omega {m_e}}}} \right)}^2}} \right]}}{\left( {\frac{e}{{i\omega {m_e}}}} \right)^2}{B_b}+\frac{{i{k_z}}}{{{\mu _0}e}}\frac{{{k_y}}}{\omega } \hfill \\ \end{gathered} \right.$$

25 $$\left\{ \begin{gathered} {A_{21}}=\frac{{{u_{b0z}}}}{{{k_z}{u_{b0z}} - \omega }}\left[ {\frac{{e{k_y}{n_{b0}}{u_{b0z}}}}{{{\gamma _0}{m_e}\left( {i\omega - i{k_z}{u_{b0z}}} \right)}}\frac{{{k_z}}}{\omega } - \frac{{e{k_y}{n_{b0}}}}{{{\gamma _0}{m_e}\left( {i\omega - i{k_z}{u_{b0z}}} \right)}}} \right] \\ - \frac{{{n_{p0}}}}{{\left[ {1+{{\left( {\frac{{e{B_b}}}{{i\omega {m_e}}}} \right)}^2}} \right]}}{\left( {\frac{e}{{i\omega {m_e}}}} \right)^2}{B_b}+\frac{{i{k_y}}}{{{\mu _0}e}}\frac{{{k_z}}}{\omega } \\ {A_{22}}=\frac{{e{n_{b0}}\left( {{{u_{{b0z}}^{2}} \mathord{\left/ {\vphantom {{u_{{b0z}}^{2}} {{C^2}}}} \right. \kern-0pt} {{C^2}}}+1} \right)}}{{{\gamma _0}{m_e}\left( {i\omega - i{k_z}{u_{b0z}}} \right)}}+\frac{{{n_{p0}}}}{{\left[ {1+{{\left( {\frac{{e{B_b}}}{{i\omega {m_e}}}} \right)}^2}} \right]}}\frac{e}{{i\omega {m_e}}}+\frac{{i\omega }}{{e{\mu _0}{C^2}}} - \frac{{i{k_y}}}{{{\mu _0}e}}\frac{{{k_y}}}{\omega } \\ - \frac{{{u_{b0z}}}}{{{k_z}{u_{b0z}} - \omega }}\left[ {\frac{{e{k_y}{n_{b0}}{u_{b0z}}}}{{{\gamma _0}{m_e}\left( {i\omega - i{k_z}{u_{b0z}}} \right)}}\frac{{{k_y}}}{\omega }+\frac{{e{k_z}{n_{b0}}\left( {{{u_{{b0z}}^{2}} \mathord{\left/ {\vphantom {{u_{{b0z}}^{2}} {{C^2}}}} \right. \kern-0pt} {{C^2}}}+1} \right)}}{{{\gamma _0}{m_e}\left( {i\omega - i{k_z}{u_{b0z}}} \right)}}} \right] \\ \end{gathered} \right.$$

To satisfy that the above Eqs. (22–23) have non-zero solutions, the determinant coefficient of the equations must be 0.

26 $$\left| {\begin{array}{*{20}{c}} A&B \\ C&D \end{array}} \right|=0$$

**Fig. 2:**
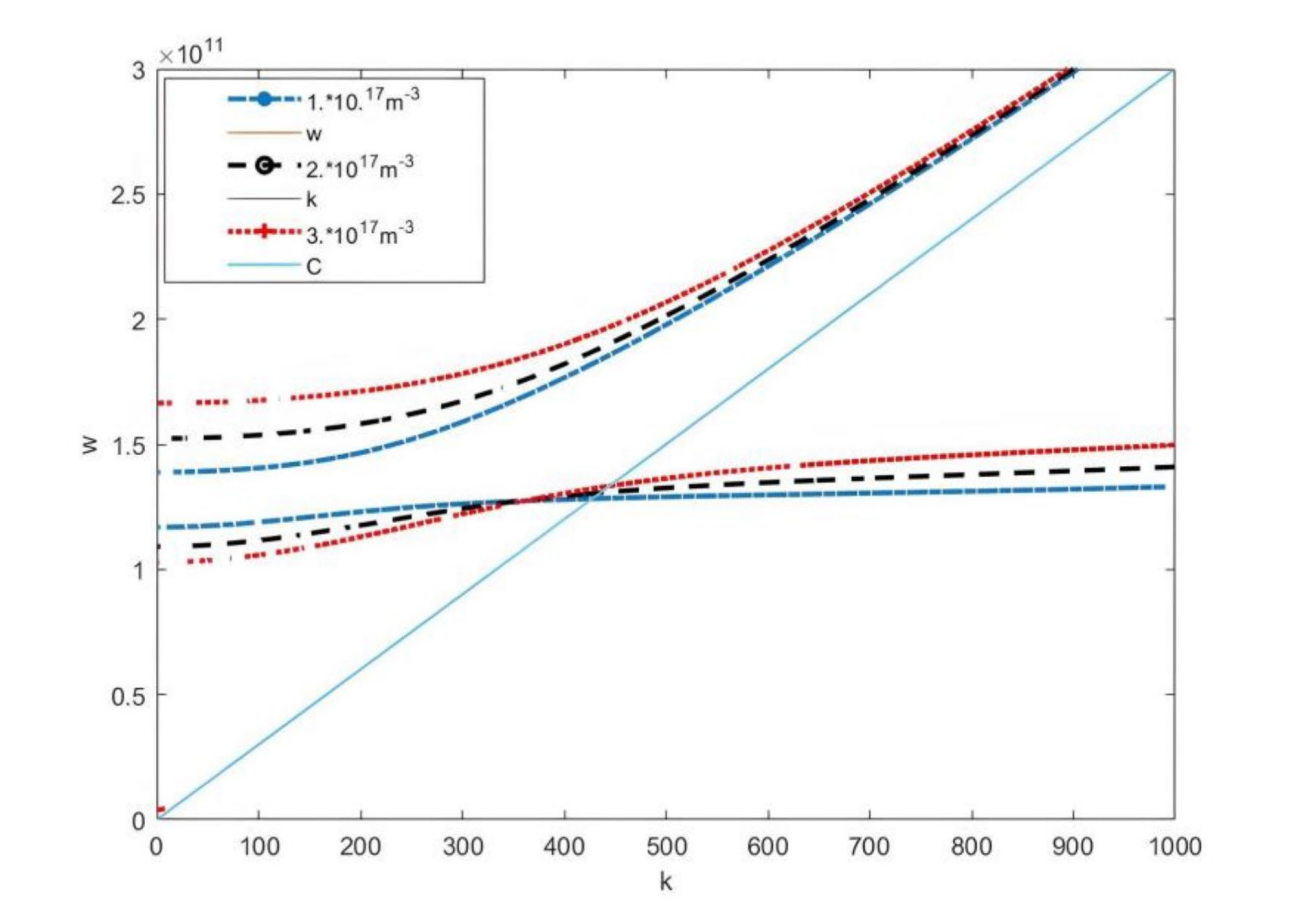
Dispersion curves under different electron beam densities (perturbation method).

Equation 26 ultimately defines the dispersion relationship of plasma microwave radiation using the perturbation method. Figure 2 illustrates the dispersion relationship between *k*_z_ and *w* under a plasma density of 5*10^18^m^−3^, an electron beam velocity of 1*10^8^m/s, and electron beam densities of 1*10^17^m^−3^,2*10^17^m^−3^,3*10^17^m^−3^, respectively. The straight line in the Fig. 2 represents the speed of light, and the non-radiative region lies to the left of this line. Our focus is on the dispersion relationship to the right side of the speed of light. As the electron beam density increases, the radiation frequency also increases accordingly.

**Fig. 3:**
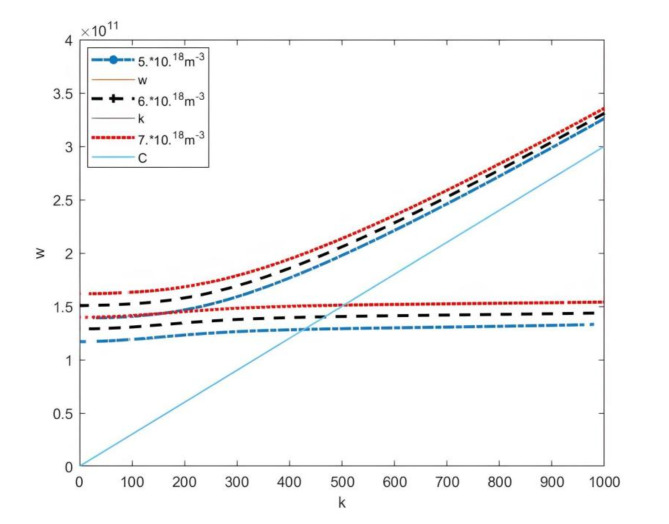
Dispersion curves under different plasma densities (perturbation method).

**Fig. 4:**
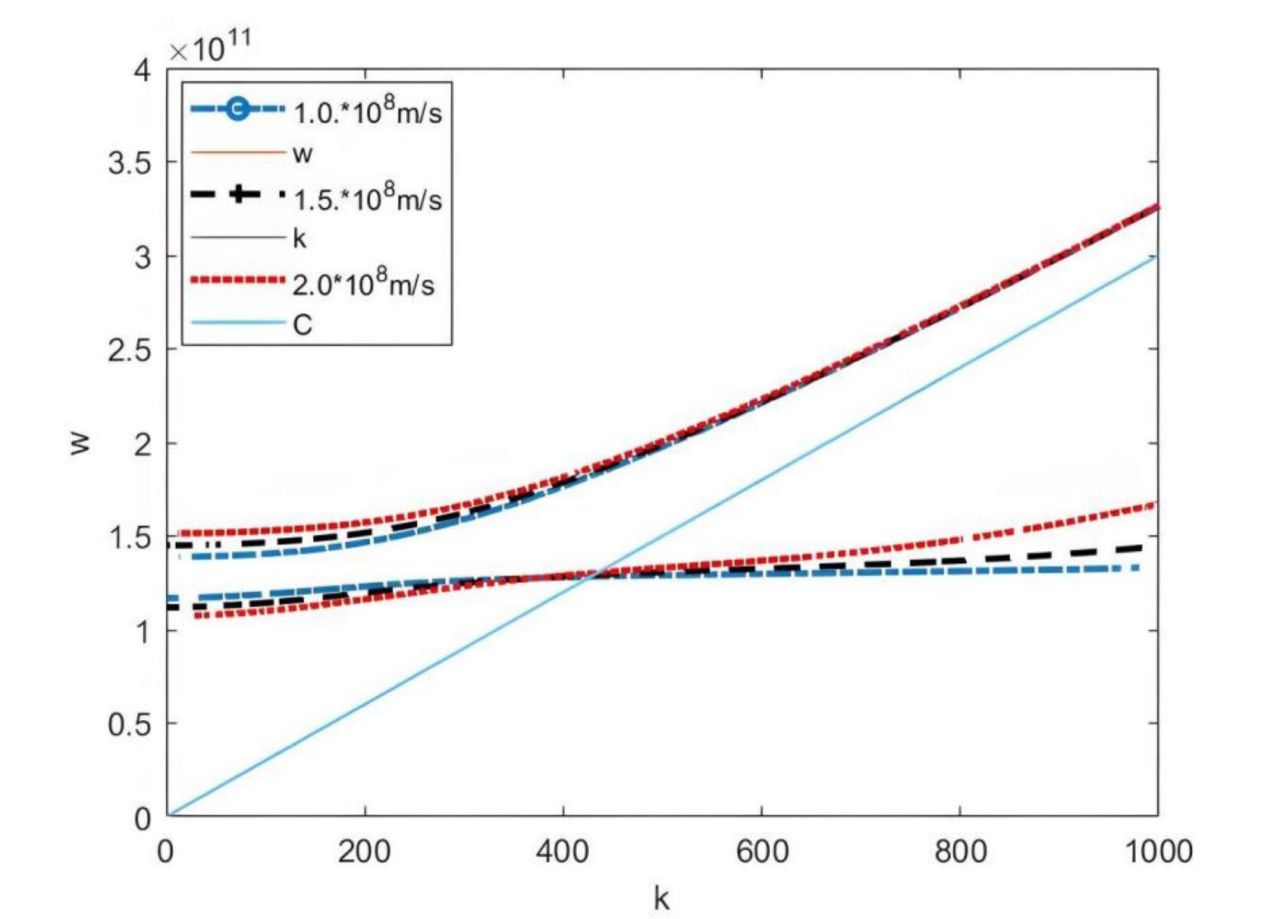
Dispersion curves under different electron beam velocity (perturbation method).

Figure 3 presents the dispersion curves at different plasma densities. As the plasma density increases, the radiation frequency also rises accordingly. During the research it was observed that if the plasma density continues to increase while the electron beam density and velocity remain constant (indicating a fixed electron beam current in the experiment), there will be no dispersion relationship when the plasma density increases to a certain threshold. This means that electromagnetic waves will not be radiated. This phenomenon suggests that if the electron beam current is too low, the electron beam will not sufficient to disturb the plasma to generate microwave radiation. The above conclusion can also be confirmed experimentally. Figure 4 illustrates the effect of different electron beam velocities on microwave radiation frequency. The higher the electron beam velocity, the higher the microwave radiation frequency.

The second part of this article uses perturbation method to derive the dispersion relationship for microwave radiation generated by the interaction between electron beam and plasma, under infinite boundary conditions. In the third part of this article, the field matching method is applied, taking into account the boundary conditions between the electron beam and plasma. The results obtained are then compared with the those from the perturbation method, and the effects of different parameters on the radiation bandwidth are further analyzed.

## Solution of dispersion relation - field - matching method

In the field matching method, the perturbation of each physical quantity is${f_1} \propto \exp \left( {i{k_z}z - i\omega t} \right)$. The equation of electron beam motion is still determined by Eqs. (6), (7). Using Eqs. 6 and 7, the perturbation density of the electron beam and the perturbation velocity of each component can be obtained.

27 $${n_{b1}}=\frac{{{n_{b0}}}}{{\omega - k{u_{b0z}}}}{k_z}{u_{bz}} - i\frac{{{n_{b0}}}}{{\omega - k{u_{b0z}}}}\frac{{\partial {u_{by}}}}{{\partial y}}$$

28 $$\left\{ \begin{gathered} {u_{by}}=\frac{e}{{{\gamma _0}{m_e}\left( {i\omega - i{k_z}{u_{b0z}}} \right)}}{E_{by}}+\frac{{e{u_{b0z}}}}{{{\gamma _0}{m_e}\left( {i\omega - i{k_z}{u_{b0z}}} \right)}}{B_{1x}} \hfill \\ {u_{bz}}=\frac{{\left( {1+\frac{{u_{{b0z}}^{2}}}{{{C^2}}}} \right)e}}{{{\gamma _0}{m_e}\left( {i\omega - i{k_z}{u_{b0z}}} \right)}}{E_{bz}} \hfill \\ \end{gathered} \right.$$

The relationship in the electromagnetic field is as follows

29 $$\nabla \times E=\frac{{\partial {E_z}}}{{\partial y}} - \frac{{\partial {E_y}}}{{\partial z}}= - \frac{{\partial {B_{1x}}}}{{\partial t}}=i\omega {B_{1x}} \Rightarrow {B_{1x}}=\frac{1}{{i\omega }}\left( {\frac{{\partial {E_{bz}}}}{{\partial y}} - \frac{{\partial {E_{by}}}}{{\partial z}}} \right)$$

We have

30 $$\left\{ \begin{gathered} {u_{by}}=\left( {\frac{e}{{{\gamma _0}{m_e}\left( {i\omega - i{k_z}{u_{b0z}}} \right)}} - \frac{{{k_z}}}{\omega }\frac{{e{u_{b0z}}}}{{{\gamma _0}{m_e}\left( {i\omega - i{k_z}{u_{b0z}}} \right)}}} \right){E_{by}}+\frac{1}{{i\omega }}\frac{{e{u_{b0z}}}}{{{\gamma _0}{m_e}\left( {i\omega - i{k_z}{u_{b0z}}} \right)}}\frac{{\partial {E_{bz}}}}{{\partial y}} \hfill \\ {u_{bz}}=\frac{{\left( {1+\frac{{u_{{b0z}}^{2}}}{{{C^2}}}} \right)e}}{{{\gamma _0}{m_e}\left( {i\omega - i{k_z}{u_{b0z}}} \right)}}{E_{bz}} \hfill \\ \end{gathered} \right.$$

Using Eq. 10, the perturbed current density of the electron beam can be obtained by combining Eqs. (27), (30)

31 $${J_{bz}}= - e{n_{b0}}{u_{bz}} - e{n_{b1}}{u_{b0z}}={C_1}{E_{bz}}+{C_2}\frac{{\partial {E_{by}}}}{{\partial y}}+{C_3}\frac{{{\partial ^2}{E_{bz}}}}{{\partial {y^2}}}$$

Where

32 $$\left\{ \begin{gathered} {C_1}= - e{n_{b0}}\frac{{\left( {1+\frac{{u_{{b0z}}^{2}}}{{{C^2}}}} \right)e}}{{{\gamma _0}{m_e}\left( {i\omega - i{k_z}{u_{b0z}}} \right)}} - \frac{{e{u_{b0z}}{k_z}{n_{b0}}}}{{\omega - k{u_{b0z}}}}\frac{{\left( {1+\frac{{u_{{b0z}}^{2}}}{{{C^2}}}} \right)e}}{{{\gamma _0}{m_e}\left( {i\omega - i{k_z}{u_{b0z}}} \right)}} \\ {C_2}=\frac{{ie{u_{b0z}}{n_{b0}}}}{{\omega - k{u_{b0z}}}}\frac{e}{{{\gamma _0}{m_e}\left( {i\omega - i{k_z}{u_{b0z}}} \right)}}\left( {1 - \frac{{{k_z}}}{\omega }{u_{b0z}}} \right) \\ {C_3}=\frac{1}{\omega }\frac{{e{u_{b0z}}{n_{b0}}}}{{\omega - k{u_{b0z}}}}\frac{{e{u_{b0z}}}}{{{\gamma _0}{m_e}\left( {i\omega - i{k_z}{u_{b0z}}} \right)}} \\ \end{gathered} \right.$$

33 $${J_{by}}= - e{n_{b0}}{u_{by}}={C_4}{E_{by}}+{C_5}\frac{{\partial {E_{bz}}}}{{\partial y}}$$

34 $$\left\{ \begin{gathered} {C_4}= - e{n_{b0}}\frac{e}{{{\gamma _0}{m_e}\left( {i\omega - i{k_z}{u_{b0z}}} \right)}}\left( {1 - {u_{b0z}}\frac{{{k_z}}}{\omega }} \right) \\ {C_5}= - \frac{1}{{i\omega }}\frac{{{e^2}{n_{b0}}{u_{b0z}}}}{{{\gamma _0}{m_e}\left( {i\omega - i{k_z}{u_{b0z}}} \right)}} \\ \end{gathered} \right.$$

Like the electron beam solution method, the perturbations of plasma electron density, electron velocity and current density can be obtained by Eqs. (4) and (5).

35 $${n_{pe}}=\frac{{{k_z}{n_{p0}}}}{\omega }{u_{pz}} - i\frac{{{n_{p0}}}}{\omega }\frac{{\partial {u_{py}}}}{{\partial y}}$$

36 $$\left\{ \begin{gathered} {u_{py}}=\frac{1}{{\left[ {1+{{\left( {\frac{{e{B_b}}}{{i\omega {m_e}}}} \right)}^2}} \right]}}\frac{e}{{i\omega {m_e}}}{E_{py}}+\frac{1}{{\left[ {1+{{\left( {\frac{{e{B_b}}}{{i\omega {m_e}}}} \right)}^2}} \right]}}{\left( {\frac{e}{{i\omega {m_e}}}} \right)^2}{B_b}{E_{pz}} \hfill \\ {u_{pz}}=\frac{1}{{\left[ {1+{{\left( {\frac{{e{B_b}}}{{i\omega {m_e}}}} \right)}^2}} \right]}}\frac{e}{{i\omega {m_e}}}{E_{pz}} - \frac{1}{{\left[ {1+{{\left( {\frac{{e{B_b}}}{{i\omega {m_e}}}} \right)}^2}} \right]}}{\left( {\frac{e}{{i\omega {m_e}}}} \right)^2}{B_b}{E_{py}} \hfill \\ \end{gathered} \right.$$

37 $$\left\{ \begin{gathered} {J_{py}}=\frac{{ - e{n_{p0}}}}{{\left[ {1+{{\left( {\frac{{e{B_b}}}{{i\omega {m_e}}}} \right)}^2}} \right]}}\frac{e}{{i\omega {m_e}}}{E_{py}}+\frac{{ - e{n_{p0}}}}{{\left[ {1+{{\left( {\frac{{e{B_b}}}{{i\omega {m_e}}}} \right)}^2}} \right]}}{\left( {\frac{e}{{i\omega {m_e}}}} \right)^2}{B_b}{E_{pz}} \hfill \\ {J_{pz}}=\frac{{ - e{n_{p0}}}}{{\left[ {1+{{\left( {\frac{{e{B_b}}}{{i\omega {m_e}}}} \right)}^2}} \right]}}\frac{e}{{i\omega {m_e}}}{E_{pz}} - \frac{{ - e{n_{p0}}}}{{\left[ {1+{{\left( {\frac{{e{B_b}}}{{i\omega {m_e}}}} \right)}^2}} \right]}}{\left( {\frac{e}{{i\omega {m_e}}}} \right)^2}{B_b}{E_{py}} \hfill \\ \end{gathered} \right.$$

${B_b}$is the magnetic field generated by the electron beam. According to Maxwell Eqs. (8) and (9), the disturbance of electric field satisfies the wave equation:

38 $$- \nabla \times \left( {\nabla \times E} \right)+\frac{{{\omega ^2}}}{{{C^2}}}E= - i\omega {\mu _0}J$$

Equation 38 can be decomposed into two directions of y and z.

39 $$- \frac{{{\omega ^2}}}{{{C^2}}}{E_y} - i\omega {\mu _0}{J_y}=\frac{\partial }{{\partial z}}\left( {\frac{{\partial {E_z}}}{{\partial y}} - \frac{{\partial {E_y}}}{{\partial z}}} \right)$$

40 $$- \frac{{{\omega ^2}}}{{{C^2}}}{E_z} - i\omega {\mu _0}{J_z}= - \frac{\partial }{{\partial y}}\left( {\frac{{\partial {E_z}}}{{\partial y}} - \frac{{\partial {E_y}}}{{\partial z}}} \right)$$

Next, the induced electric field of the inside the electron beam is solved. Bring Eq. (33) into Eq. (39), we obtain the relationship between ${E_{by}}$ and ${E_{bz}}$

41 $${E_{by}}={C_6}\frac{{\partial {E_{bz}}}}{{\partial y}}$$

Where

42 $${C_6}=\frac{{i{k_z}+i\omega {\mu _0}{C_5}}}{{ - \frac{{{\omega ^2}}}{{{C^2}}} - i\omega {\mu _0}{C_4} - k_{z}^{2}}}$$

Bring Eqs. (41) and (31) into Eq. (40), we obtain

43 $${C_7}{E_{bz}}+{C_8}\frac{{{\partial ^2}{E_{bz}}}}{{\partial {y^2}}}=0$$

Where

44 $$\left\{ \begin{gathered} {C_7}=\frac{{{\omega ^2}}}{{{C^2}}}+i\omega {\mu _0}{C_1} \hfill \\ {C_8}=ik{C_6}+i\omega {\mu _0}{C_3}+i\omega {\mu _0}{C_2}{C_6} - 1 \hfill \\ \end{gathered} \right.$$

Solve Eq. (43)

45 $${E_{bz}}={f_1}\sin \left( {my} \right)+{f_2}\cos \left( {my} \right)$$

46 $$m=\sqrt {{{{C_8}} \mathord{\left/ {\vphantom {{{C_8}} {{C_7}}}} \right. \kern-0pt} {{C_7}}}}$$

Bring Eq. (45) into Eq. (41), we obtain

47 $${E_{by}}={C_6}m{f_1}\cos \left( {my} \right) - {C_6}m{f_2}\sin \left( {my} \right)$$

Using a similar method, we can calculate the intensity of the induced electric field in the plasma region. Bring Eq. (37) into Eq. (39), we obtain the relationship between ${E_{py}}$ and ${E_{pz}}$

48 $${E_{py}}={C_9}{E_{pz}}+{C_{10}}\frac{{\partial {E_{pz}}}}{{\partial y}}$$

Where

49 $$\left\{ \begin{gathered} {C_9}= - \frac{1}{{\frac{{i\omega {\mu _0}e{n_{p0}}}}{{\left[ {1+{{\left( {\frac{{e{B_b}}}{{i\omega {m_e}}}} \right)}^2}} \right]}}\frac{e}{{i\omega {m_e}}} - \frac{{{\omega ^2}}}{{{C^2}}} - k_{z}^{2}}}\frac{{i\omega {\mu _0}e{n_{p0}}}}{{\left[ {1+{{\left( {\frac{{e{B_b}}}{{i\omega {m_e}}}} \right)}^2}} \right]}}{\left( {\frac{e}{{i\omega {m_e}}}} \right)^2}{B_b} \hfill \\ {C_{10}}=\frac{{i{k_z}}}{{\frac{{i\omega {\mu _0}e{n_{p0}}}}{{\left[ {1+{{\left( {\frac{{e{B_b}}}{{i\omega {m_e}}}} \right)}^2}} \right]}}\frac{e}{{i\omega {m_e}}} - \frac{{{\omega ^2}}}{{{C^2}}} - k_{z}^{2}}} \hfill \\ \end{gathered} \right.$$

Bring Eqs. (48) and (37) into Eq. (40), we obtain

50 $$\frac{{{\partial ^2}{E_{pz}}}}{{\partial {y^2}}}+{C_{11}}\frac{{\partial {E_{pz}}}}{{\partial y}}+{C_{12}}{E_{pz}}=0$$

Where

51 $$\left\{ \begin{gathered} {C_{11}}=\frac{1}{{i{k_z}{C_{10}} - 1}}\left( {{C_{10}}\frac{{i\omega {\mu _0}e{n_{p0}}}}{{\left[ {1+{{\left( {\frac{{e{B_b}}}{{i\omega {m_e}}}} \right)}^2}} \right]}}{{\left( {\frac{e}{{i\omega {m_e}}}} \right)}^2}{B_b}+i{k_z}{C_9}} \right) \hfill \\ {C_{12}}=\frac{1}{{i{k_z}{C_{10}} - 1}}\left( {\frac{{{\omega ^2}}}{{{C^2}}}+\frac{{ - {\mu _0}e{n_{p0}}}}{{\left[ {1+{{\left( {\frac{{e{B_b}}}{{i\omega {m_e}}}} \right)}^2}} \right]}}\frac{e}{{{m_e}}}+{C_9}\frac{{i\omega {\mu _0}e{n_{p0}}}}{{\left[ {1+{{\left( {\frac{{e{B_b}}}{{i\omega {m_e}}}} \right)}^2}} \right]}}{{\left( {\frac{e}{{i\omega {m_e}}}} \right)}^2}{B_b}} \right) \hfill \\ \end{gathered} \right.$$

Solve Eq. (50)

52 $${E_{pz}}={g_1}\exp \left( {{n_1}y} \right)+{g_2}\exp \left( {{n_2}y} \right)$$

Where

53 $${n_{1,2}}=\frac{{ - {C_{11}} \pm \sqrt {C_{{11}}^{2} - 4{C_{12}}} }}{2}$$

Bring Eq. (52) into Eq. (48), we obtain

54 $${E_{py}}=\left[ {{C_9}+{C_{10}}{n_1}} \right]{g_1}\exp \left( {{n_1}y} \right)+\left[ {{C_9}+{C_{10}}{n_2}} \right]{g_2}\exp \left( {{n_2}y} \right)$$

Using the boundary conditions of electromagnetic field, the dispersion relation between electron beam and transverse magnetized plasma can be obtained. At the boundary between electron beam and plasma, $y={y_0}$ we obtain

55 $${B_{bx}}={B_{px}}$$

56 $${B_{bz}} - {B_{pz}}={J_{py}}+{J_{by}}$$

57 $${E_{bz}}={E_{pz}}$$

58 $$\int {D \cdot ds} =\int {\delta \rho } dV= - e\int {{n_1}dV}$$

Using Eq. 8, simultaneous Eqs. (45), (47), (52), (54), We obtain

59 $${B_z}=0$$

60 $${B_{bx}}=\frac{1}{{i\omega }}\left[ {{f_1}m - i{k_z}{C_6}{f_1}m} \right]\cos \left( {my} \right)+\frac{1}{{i\omega }}\left[ {i{k_z}{C_6}m{f_2} - m{f_2}} \right]\sin \left( {my} \right)$$

61 $${B_{px}}=\frac{1}{{i\omega }}\left[ {{g_1}{n_1} - i{k_z}\left[ {{C_9}+{C_{10}}{n_1}} \right]{g_1}} \right]\exp \left( {{n_1}y} \right)+\frac{1}{{i\omega }}\left[ {{g_2}{n_2} - i{k_z}\left[ {{C_9}+{C_{10}}{n_2}} \right]{g_2}} \right]\exp \left( {{n_2}y} \right)$$

Using Eq. 55, simultaneous Eqs. (60), (61), We obtain

62 $$\begin{gathered} {f_1}\left[ {m - i{k_z}{C_6}m} \right]\cos \left( {m{y_0}} \right)+{f_2}\left[ {i{k_z}{C_6}m - m} \right]\sin \left( {m{y_0}} \right)= \hfill \\ {g_1}\left[ {{n_1} - i{k_z}{C_9} - i{k_z}{C_{10}}{n_1}} \right]\exp \left( {{n_1}{y_0}} \right)+{g_2}\left[ {{n_2} - i{k_z}{C_9} - i{k_z}{C_{10}}{n_2}} \right]\exp \left( {{n_2}{y_0}} \right) \hfill \\ \end{gathered}$$

Substitute Eqs. 45,47 and 52, 54 into Eqs. 33, 37 respectively, and use boundary condition 56 to obtain

63 $$\begin{gathered} {g_1}\left[ {\frac{{ - e{n_{p0}}}}{{\left[ {1+{{\left( {\frac{{e{B_b}}}{{i\omega {m_e}}}} \right)}^2}} \right]}}\frac{e}{{i\omega {m_e}}}\left[ {{C_9}+{C_{10}}{n_1}} \right]+\frac{{ - e{n_{p0}}}}{{\left[ {1+{{\left( {\frac{{e{B_b}}}{{i\omega {m_e}}}} \right)}^2}} \right]}}{{\left( {\frac{e}{{i\omega {m_e}}}} \right)}^2}{B_b}} \right]\exp \left( {{n_1}{y_0}} \right) \hfill \\ +{g_2}\left[ {\frac{{ - e{n_{p0}}}}{{\left[ {1+{{\left( {\frac{{e{B_b}}}{{i\omega {m_e}}}} \right)}^2}} \right]}}\frac{e}{{i\omega {m_e}}}\left[ {{C_9}+{C_{10}}{n_2}} \right]+\frac{{ - e{n_{p0}}}}{{\left[ {1+{{\left( {\frac{{e{B_b}}}{{i\omega {m_e}}}} \right)}^2}} \right]}}{{\left( {\frac{e}{{i\omega {m_e}}}} \right)}^2}{B_b}} \right]\exp \left( {{n_2}{y_0}} \right) \hfill \\ +{f_1}\left[ {{C_4}{C_6}m+{C_5}m} \right]\cos \left( {m{y_0}} \right) - {f_2}\left[ {{C_5}m+{C_4}{C_6}m} \right]\sin \left( {m{y_0}} \right)=0 \hfill \\ \end{gathered}$$

Using Eq. 57, We obtain

64 $${g_1}\exp \left( {{n_1}{y_0}} \right)+{g_2}\exp \left( {{n_2}{y_0}} \right) - {f_1}\sin \left( {m{y_0}} \right) - {f_2}\cos \left( {m{y_0}} \right)=0$$

The perturbation density ${n_1}$ (Eq. 58) is the sum of perturbation densities ${n_{b1}}$ and ${n_{p1}}$ on both sides outside the electron beam and inside the electron beam, ${n_1}={n_{b1}}+{n_{p1}}$, using Eqs. (27) and (35)

65 $${n_1}= - i\frac{{{n_{b0}}}}{{\omega - k{u_{b0z}}}}\frac{{\partial {u_{bz}}}}{{\partial z}} - i\frac{{{n_{b0}}}}{{\omega - k{u_{b0z}}}}\frac{{\partial {u_{by}}}}{{\partial y}} - i\frac{{{n_{p0}}}}{\omega }\frac{{\partial {u_{pz}}}}{{\partial z}} - i\frac{{{n_{p0}}}}{\omega }\frac{{\partial {u_{py}}}}{{\partial y}}$$

When $y \to {y_0}$ bring (65) into (58), we have

66 $${E_{by}} - {E_{py}}=i\frac{{e{n_{b0}}}}{{{\varepsilon _0}\left( {\omega - k{u_{b0z}}} \right)}}{u_{bz}}+i\frac{{e{n_{b0}}}}{{{\varepsilon _0}\left( {\omega - k{u_{b0z}}} \right)}}{u_{by}}+i\frac{{e{n_{p0}}}}{{{\varepsilon _0}\omega }}{u_{pz}}+i\frac{{e{n_{p0}}}}{{{\varepsilon _0}\omega }}{u_{py}}$$

Bring Eqs. (47), (54), (30), (36) into (66), we obtain

67 $${C_{13}}{f_1}+{C_{14}}{f_2}+{C_{15}}{g_1}+{C_{16}}{g_2}=0$$

Where

68 $$\begin{gathered} {C_{13}}=i\frac{{e{n_{b0}}}}{{{\varepsilon _0}\left( {\omega - k{u_{b0z}}} \right)}}\frac{{\left( {1+\frac{{u_{{b0z}}^{2}}}{{{C^2}}}} \right)e}}{{{\gamma _0}{m_e}\left( {i\omega - i{k_z}{u_{b0z}}} \right)}}\sin \left( {m{y_0}} \right)+\frac{{e{n_{b0}}}}{{\omega {\varepsilon _0}\left( {\omega - k{u_{b0z}}} \right)}}\frac{{e{u_{b0z}}}}{{{\gamma _0}{m_e}\left( {i\omega - i{k_z}{u_{b0z}}} \right)}}m\cos \left( {m{y_0}} \right) \\ +\left[ {i\frac{{e{n_{b0}}}}{{{\varepsilon _0}\left( {\omega - k{u_{b0z}}} \right)}}\left( {\frac{e}{{{\gamma _0}{m_e}\left( {i\omega - i{k_z}{u_{b0z}}} \right)}} - \frac{{{k_z}}}{\omega }\frac{{e{u_{b0z}}}}{{{\gamma _0}{m_e}\left( {i\omega - i{k_z}{u_{b0z}}} \right)}}} \right) - 1} \right]{C_6}m\cos \left( {m{y_0}} \right) \\ \end{gathered}$$

69 $$\begin{gathered} {C_{14}}=i\frac{{e{n_{b0}}}}{{{\varepsilon _0}\left( {\omega - k{u_{b0z}}} \right)}}\frac{{\left( {1+\frac{{u_{{b0z}}^{2}}}{{{C^2}}}} \right)e}}{{{\gamma _0}{m_e}\left( {i\omega - i{k_z}{u_{b0z}}} \right)}}\cos \left( {m{y_0}} \right) - \frac{{e{n_{b0}}}}{{\omega {\varepsilon _0}\left( {\omega - k{u_{b0z}}} \right)}}\frac{{e{u_{b0z}}}}{{{\gamma _0}{m_e}\left( {i\omega - i{k_z}{u_{b0z}}} \right)}}m\sin \left( {m{y_0}} \right) \\ - \left[ {i\frac{{e{n_{b0}}}}{{{\varepsilon _0}\left( {\omega - k{u_{b0z}}} \right)}}\left( {\frac{e}{{{\gamma _0}{m_e}\left( {i\omega - i{k_z}{u_{b0z}}} \right)}} - \frac{{{k_z}}}{\omega }\frac{{e{u_{b0z}}}}{{{\gamma _0}{m_e}\left( {i\omega - i{k_z}{u_{b0z}}} \right)}}} \right) - 1} \right]{C_6}m\sin \left( {m{y_0}} \right) \\ \end{gathered}$$

70 $$\begin{gathered} {C_{15}}=\left[ {i\frac{{e{n_{p0}}}}{{{\varepsilon _0}\omega }}\frac{1}{{\left[ {1+{{\left( {\frac{{e{B_b}}}{{i\omega {m_e}}}} \right)}^2}} \right]}}\frac{e}{{i\omega {m_e}}}+i\frac{{e{n_{p0}}}}{{{\varepsilon _0}\omega }}\frac{1}{{\left[ {1+{{\left( {\frac{{e{B_b}}}{{i\omega {m_e}}}} \right)}^2}} \right]}}{{\left( {\frac{e}{{i\omega {m_e}}}} \right)}^2}{B_b}} \right]\exp \left( {{n_1}{y_0}} \right) \\ +\left[ {i\frac{{e{n_{p0}}}}{{{\varepsilon _0}\omega }}\frac{1}{{\left[ {1+{{\left( {\frac{{e{B_b}}}{{i\omega {m_e}}}} \right)}^2}} \right]}}\frac{e}{{i\omega {m_e}}} - i\frac{{e{n_{p0}}}}{{{\varepsilon _0}\omega }}\frac{1}{{\left[ {1+{{\left( {\frac{{e{B_b}}}{{i\omega {m_e}}}} \right)}^2}} \right]}}{{\left( {\frac{e}{{i\omega {m_e}}}} \right)}^2}{B_b}+1} \right]\left[ {{C_9}+{C_{10}}{n_1}} \right]\exp \left( {{n_1}{y_0}} \right) \\ \end{gathered}$$

71 $$\begin{gathered} {C_{16}}=\left[ {i\frac{{e{n_{p0}}}}{{{\varepsilon _0}\omega }}\frac{1}{{\left[ {1+{{\left( {\frac{{e{B_b}}}{{i\omega {m_e}}}} \right)}^2}} \right]}}\frac{e}{{i\omega {m_e}}}+i\frac{{e{n_{p0}}}}{{{\varepsilon _0}\omega }}\frac{1}{{\left[ {1+{{\left( {\frac{{e{B_b}}}{{i\omega {m_e}}}} \right)}^2}} \right]}}{{\left( {\frac{e}{{i\omega {m_e}}}} \right)}^2}{B_b}} \right]\exp \left( {{n_2}{y_0}} \right) \\ +\left[ {i\frac{{e{n_{p0}}}}{{{\varepsilon _0}\omega }}\frac{1}{{\left[ {1+{{\left( {\frac{{e{B_b}}}{{i\omega {m_e}}}} \right)}^2}} \right]}}\frac{e}{{i\omega {m_e}}} - i\frac{{e{n_{p0}}}}{{{\varepsilon _0}\omega }}\frac{1}{{\left[ {1+{{\left( {\frac{{e{B_b}}}{{i\omega {m_e}}}} \right)}^2}} \right]}}{{\left( {\frac{e}{{i\omega {m_e}}}} \right)}^2}{B_b}+1} \right]\left[ {{C_9}+{C_{10}}{n_2}} \right]\exp \left( {{n_2}{y_0}} \right) \\ \end{gathered}$$

Equations (62), (63),(64),(67) constitutes an equation system with respect to variable ${k_z}$and$\omega$. If the equation system has a solution, its coefficient determinant is 0. Simplify the above Eqs. (62), (63), (64),(67), we obtain

72 $$\left\{ \begin{gathered} {a_{11}}{f_1}+{a_{12}}{f_2}+{a_{13}}{g_1}+{a_{14}}{g_2}=0 \hfill \\ {a_{21}}{f_1}+{a_{22}}{f_2}+{a_{23}}{g_1}+{a_{24}}{g_2}=0 \hfill \\ {a_{31}}{f_1}+{a_{32}}{f_2}+{a_{33}}{g_1}+{a_{34}}{g_2}=0 \hfill \\ {a_{41}}{f_1}+{a_{42}}{f_2}+{a_{43}}{g_1}+{a_{44}}{g_2}=0 \hfill \\ \end{gathered} \right.$$

And the dispersion relation is in the following form

73 $$\left| {\begin{array}{*{20}{c}} {{a_{11}}}&{{a_{12}}}&{{a_{13}}}&{{a_{14}}} \\ {{a_{21}}}&{{a_{22}}}&{{a_{23}}}&{{a_{24}}} \\ {{a_{31}}}&{{a_{32}}}&{{a_{33}}}&{{a_{34}}} \\ {{a_{41}}}&{{a_{42}}}&{{a_{43}}}&{{a_{44}}} \end{array}} \right|=0$$

Where

74 $$\left\{ \begin{gathered} {a_{11}}=\left[ {m - i{k_z}{C_6}m} \right]\cos \left( {m{y_0}} \right) \hfill \\ {a_{12}}=\left[ {i{k_z}{C_6}m - m} \right]\sin \left( {m{y_0}} \right) \hfill \\ {a_{13}}= - \left[ {{n_1} - i{k_z}{C_9} - i{k_z}{C_{10}}{n_1}} \right]\exp \left( {{n_1}{y_0}} \right) \hfill \\ {a_{14}}= - \left[ {{n_2} - i{k_z}{C_9} - i{k_z}{C_{10}}{n_2}} \right]\exp \left( {{n_2}{y_0}} \right) \hfill \\ \end{gathered} \right.$$

75 $$\left\{ \begin{gathered} {a_{21}}=\left[ {{C_4}{C_6}m+{C_5}m} \right]\cos \left( {m{y_0}} \right) \hfill \\ {a_{22}}= - \left[ {{C_5}m+{C_4}{C_6}m} \right]\sin \left( {m{y_0}} \right) \hfill \\ {a_{23}}=\left[ {\frac{{ - e{n_{p0}}}}{{\left[ {1+{{\left( {\frac{{e{B_b}}}{{i\omega {m_e}}}} \right)}^2}} \right]}}\frac{e}{{i\omega {m_e}}}\left[ {{C_9}+{C_{10}}{n_1}} \right]+\frac{{ - e{n_{p0}}}}{{\left[ {1+{{\left( {\frac{{e{B_b}}}{{i\omega {m_e}}}} \right)}^2}} \right]}}{{\left( {\frac{e}{{i\omega {m_e}}}} \right)}^2}{B_b}} \right]\exp \left( {{n_1}{y_0}} \right) \hfill \\ {a_{24}}=\left[ {\frac{{ - e{n_{p0}}}}{{\left[ {1+{{\left( {\frac{{e{B_b}}}{{i\omega {m_e}}}} \right)}^2}} \right]}}\frac{e}{{i\omega {m_e}}}\left[ {{C_9}+{C_{10}}{n_2}} \right]+\frac{{ - e{n_{p0}}}}{{\left[ {1+{{\left( {\frac{{e{B_b}}}{{i\omega {m_e}}}} \right)}^2}} \right]}}{{\left( {\frac{e}{{i\omega {m_e}}}} \right)}^2}{B_b}} \right]\exp \left( {{n_2}{y_0}} \right) \hfill \\ \end{gathered} \right.$$

76 $$\left\{ \begin{gathered} {a_{31}}= - \sin \left( {m{y_0}} \right) \hfill \\ {a_{32}}= - \cos \left( {m{y_0}} \right) \hfill \\ {a_{33}}=\exp \left( {{n_1}{y_0}} \right) \hfill \\ {a_{34}}=\exp \left( {{n_2}{y_0}} \right) \hfill \\ \end{gathered} \right.$$

77 $$\left\{ \begin{gathered} {a_{41}}={C_{13}} \hfill \\ {a_{42}}={C_{14}} \hfill \\ {a_{43}}={C_{15}} \hfill \\ {a_{44}}={C_{16}} \hfill \\ \end{gathered} \right.$$

**Fig. 5:**
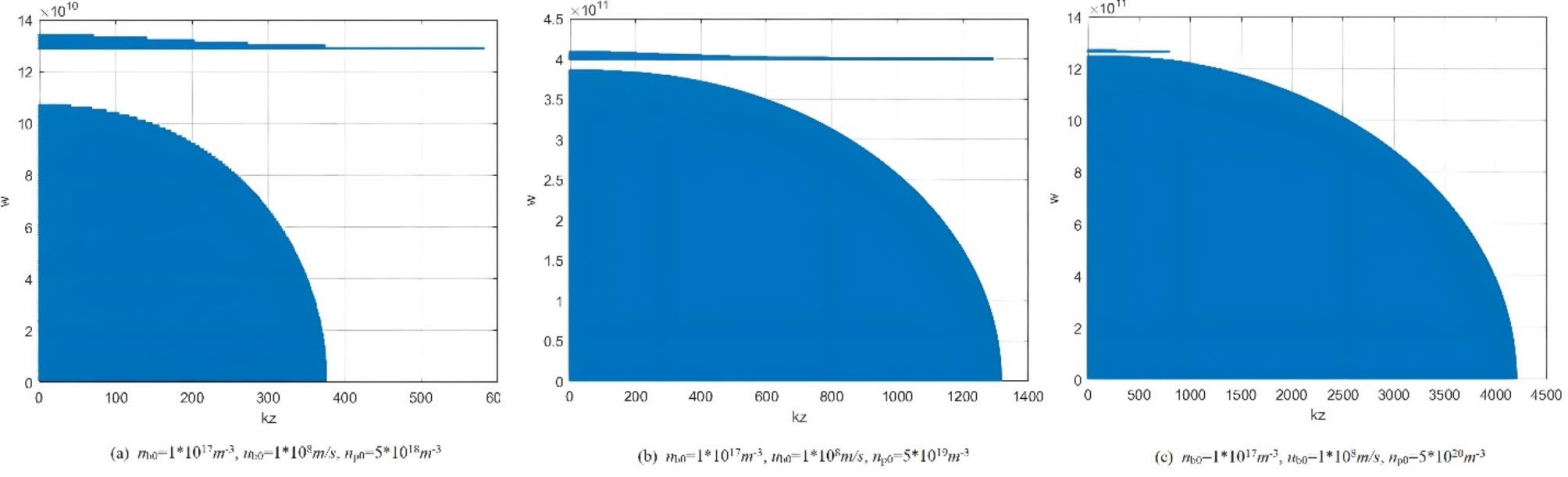
The influence of different plasma densities on dispersion relationship.

Equation (73) finally forms the final dispersion relation for microwave radiation generated by the interaction between electron beam and plasma (using the field-matching method). This equation describes the influence of various parameters on the dispersion relation. The resulting dispersion curve not only accounts for waves propagating perpendicular to the magnetic field but also includes the effects generated by axial electron beams. Figure 5 illustrates the dispersion relation of plasma radiation under the following conditions: electron beam density of 1*10^17^m^−3^, electron beam speed of 1*10^8^m/s, and plasma densities of 5*10^18^m^−3^, 5*10^19^m^−3^, 5*10^20^m^−3^ respectively. In the figure, the X-axis represents the wave number, and the Y-axis is the radiation frequency. When k = 0, w is the cutoff frequency of the wave. In the figure, the dispersion relation in the figure includes two parts: the low-frequency and high-frequency radiation bandwidths. As plasma density increases, the maximum frequency corresponding to the two radiation bandwidths also increase accordingly, which is consistent with the analysis results in Fig. 3. Additionally, as plasma density increases, the radiation bandwidth of the high-frequency part narrows, while the low-frequency radiation bandwidth increases. Typically, we focus on the high-frequency the radiation region. When the plasma density reaches a certain threshold, the radiation bandwidth of the high-frequency part vanishes, which is consistent with the conclusion of perturbation analysis.

**Fig. 6:**
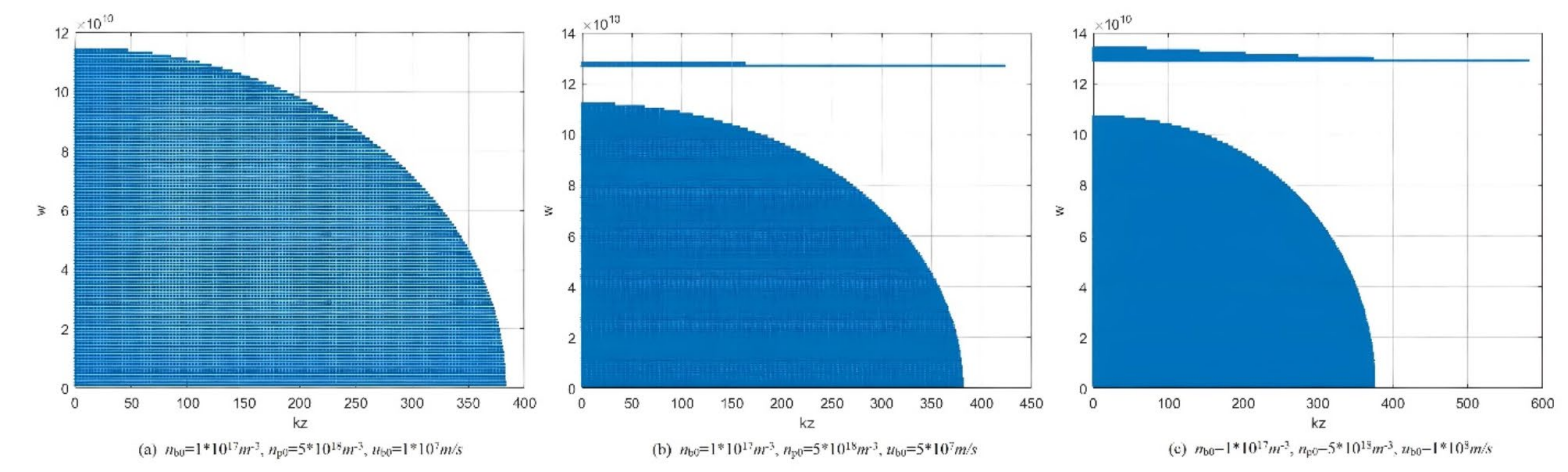
The influence of different electron beam speed on dispersion relationship.

**Fig. 7:**
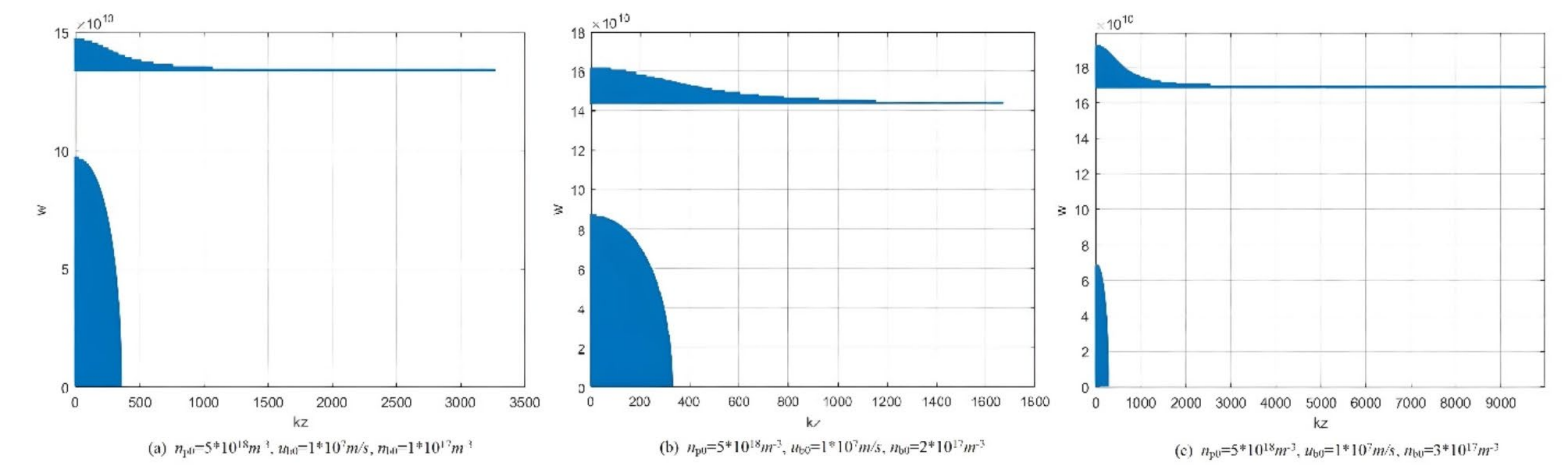
The influence of different electron beam density on dispersion relationship.

Figure 6 shows the effect of different electron beam speed on the dispersion curve. Where plasma density 5*10^18^m^−3^, electron beam density 1*10^17^m^−3^, electron beam speed 1*10^7^m/s, 5*10^7^m/s, 1*10^8^m/s, respectively. At low electron beam speeds, high-frequency radiation does not occur in the microwave radiation region. As the electron beam speed increases, the radiation bandwidth in the high-frequency region significantly increases, while the radiation frequency and radiation bandwidth in the low-frequency region remain almost unchanged. Figure 7 shows the effect of different electron beam density on the dispersion curve. Where plasma density 5*10^18^m^−3^, electron beam speed 1*10^8^m/s, electron beam density 1*10^17^m^−3^, 2*10^17^m^−3^, 3*10^17^m^−3^, respectively. As the electron beam density increases, the radiation frequency and bandwidth in the high-frequency region significantly increase. (This result is consistent with the conclusion in Fig. 2.) Based on this characteristic, in microwave radiation experiments involving the interaction between electron beams and plasma, changing the electron beam density can generate broadband microwave radiation with high radiation frequency.

## Conclusion

This article establishes a mathematical model for the interaction between electron beams and plasma and derives the dispersion relationship for microwave radiation generated by this interaction using both the perturbation method and the field matching method. During the process of high-energy electron beams passing through plasma, the electrons in the electron beam are affected by the plasma, while the electron beam simultaneously transfers energy to the electromagnetic waves generated by the interaction. The conclusions from both methods indicate that increasing electron beam density, electron beam velocity, and plasma density can lead to an increase in microwave radiation frequency. In addition, increasing the electron beam density and velocity can increase the radiation bandwidth. However, it the electron beam speed is too low, or the electron beam density is significantly lower than the plasma density, high-frequency microwave radiation cannot be generated.

## Data Availability

Data availability-All data generated or analysed during this study are included in this published article.
